# Bio-Derived Cellulose Nanofibers for the Development Under Environmentally Assessed Conditions of Cellulose/ZnO Nanohybrids with Enhanced Biocompatibility and Antimicrobial Properties

**DOI:** 10.3390/ma19020346

**Published:** 2026-01-15

**Authors:** Kyriaki Marina Lyra, Aggeliki Papavasiliou, Caroline Piffet, Lara Gumusboga, Jean-Michel Thomassin, Yana Marie, Alexandre Hoareau, Vincent Moulès, Javier Alcodori, Pau Camilleri Lledó, Albany Milena Lozano Násner, Jose Gallego, Elias Sakellis, Fotios K. Katsaros, Dimitris Tsiourvas, Zili Sideratou

**Affiliations:** 1 Institute of Nanoscience and Nanotechnology, National Center for Scientific Research ‘‘Demokritos”, 15310 Aghia Paraskevi, Greece; k.lyra@inn.demokritos.gr (K.M.L.); a.papavasiliou@inn.demokritos.gr (A.P.); or e_sakel@phys.uoa.gr (E.S.); f.katsaros@inn.demokritos.gr (F.K.K.); d.tsiourvas@inn.demokritos.gr (D.T.); 2 Celabor, Research Center, Avenue du Parc 38, 4650 Chaineux, Belgium; caroline.piffet@celabor.be (C.P.); lara.gumusboga@celabor.be (L.G.); jean-michel.thomassin@celabor.be (J.-M.T.); 3 VirHealth, Bioserra 3, 76 rue Georges Gouy, 69007 Lyon, France; y.marie@virhealth.fr (Y.M.); a.hoareau@virhealth.fr (A.H.); v.moules@virhealth.fr (V.M.); 4 Research Centre of Packaging, Transport and Logistics, ITENE, Carrer d’Albert Einstein 1, 46980 Paterna, Spain; javier.alcodori@itene.com (J.A.); pau.camilleri@itene.com (P.C.L.); 5 Association for Research and Development of Innovations and Technologies for the Protection of Environmental, Social, and Cultural Heritage, 48 Avenue Mont-Rabeau, 06200 Nice, France; milena.nasner@arditec.net (A.M.L.N.); jose.gallego@arditec.net (J.G.); 6 Condensed Matter Physics Section, Physics Department, National and Kapodistrian University of Athens, Panepistimiopolis, Zografos, 15784 Athens, Greece

**Keywords:** cellulose nanofibers, ZnO nanoparticles, antibacterial properties, antiviral properties, life cycle analysis

## Abstract

The development of eco-friendly antimicrobial materials is essential for addressing antibiotic resistance, while reducing environmental impact. In this study, bio-derived anionic and cationic cellulose nanofibers (a-CNF and c-CNF) were employed as templating matrices for the in situ hydrothermal synthesis of cellulose/ZnO nanohybrids. Physicochemical characterization confirmed efficient cellulose functionalization and high-quality nanofibrillation, as well as the formation of uniformly dispersed ZnO nanoparticles (≈10–20 nm) strongly integrated within the cellulose network. The ZnO content was 30 and 20 wt. % for a-CNF/ZnO and c-CNF/ZnO, respectively. Antibacterial evaluation against *Escherichia coli* and *Staphylococcus aureus* revealed enhanced activity for both hybrids, with c-CNF/ZnO displaying the lowest MIC/MBC values (50/100 μg/mL). Antiviral assays revealed complete feline calicivirus inactivation at 100 μg/mL for c-CNF/ZnO, while moderate activity was observed against bovine coronavirus, highlighting the role of surface charge. Cytotoxicity assays on mammalian cells demonstrated high biocompatibility at antimicrobial concentrations. Life cycle assessment showed that c-CNF/ZnO exhibits a lower overall environmental burden than a-CNF/ZnO, with electricity demand being the main contributor, indicating clear opportunities for further reductions through process optimization and scale-up. Overall, these results demonstrate that CNF/ZnO nanohybrids effectively combine renewable biopolymers with ZnO antimicrobial functionality, offering a sustainable and safe platform for biomedical and environmental applications.

## 1. Introduction

The emergence and rapid spread of antibiotic-resistant pathogens such as bacteria, fungi, and viruses have become a major global health concern [1,2]. The World Health Organization (WHO) has identified antibiotic resistance as one of the top three public health threats of the 21st century [3]. It is estimated that multidrug-resistant bacterial infections currently result in approximately 700,000 deaths worldwide each year. If effective measures are not implemented, this number is expected to increase to 10 million deaths annually by 2050, surpassing the current global mortality rate from cancer [4]. Thus, alternative antibiotic-free antimicrobial strategies based on new materials that are effective, biocompatible and environmentally sustainable are mandatory [5,6,7,8,9]. In this respect, among emerging solutions, nanostructured materials have shown remarkable potential owing to their high surface area, tunable surface chemistry, and ability to interact directly with microbial membranes [10,11]. In particular, inorganic metal and metal oxide nanoparticles (NPs) and biopolymer-based hybrid nanomaterials have intensively studied as next-generation antimicrobial systems [5,12,13,14,15,16].

Cellulose nanofibers (CNFs), derived from cellulose, the most abundant biopolymer, consist of interconnected fibrils composed of both crystalline and amorphous regions, with widths typically ranging from 5 to 30 nm and aspect ratios greater than 50 [17]. In recent years, CNFs have attracted significant attention due to their availability from sustainable and renewable resources, as well as their remarkable combination of properties, including biocompatibility, biodegradability, high mechanical strength and stiffness [18]. Owing to their large specific surface area and abundant hydroxyl groups, CNF can be readily chemically modified, leading to the formation of novel nanomaterials with enhanced properties such as UV-blocking ability, low toxicity, and antimicrobial efficacy [19,20,21,22]. Moreover, CNF can serve as excellent supports for inorganic nanoparticles, improving their properties, including dispersibility, chemical stability, and antimicrobial activity [20,22]. In addition, CNFs are inherently biocompatible and non-toxic, making them suitable for biomedical and environmental applications, including drug delivery, tissue engineering scaffolds, wound dressing, antimicrobial materials, and water treatment [17,18,23].

Among all metal oxide nanoparticles studied to date, zinc oxide (ZnO) nanoparticles are recognized for their broad-spectrum antibacterial activities, as well as for their remarkable chemical stability [24,25,26]. ZnO in its bulk form is classified as a safe material by the U.S. Food and Drug Administration (21 CFR 182.8991) and is widely utilized in various fields, including the food industry, cosmetics, textiles, wound dressings, water treatment systems, agriculture, biomedical applications, etc. [27,28,29,30,31,32]. Although extensively used, its antibacterial mechanism is not fully understood and is generally attributed to electrostatic interactions with bacterial membranes, generation of reactive oxygen species (ROS), and release of Zn^2+^ ions [33,34,35,36]. Beyond their antibacterial activities, ZnO NPs have also demonstrated promising antiviral properties [37,38,39,40,41,42,43]. Their antiviral mechanisms involve the generation of ROS that damage viral proteins and nucleic acids, direct interactions with viral surface proteins that prevent viral attachment and entry, and the disruption of viral replication processes [44,45]. Moreover, ZnO NPs can modulate host immune responses, enhancing antiviral defense. Several studies have reported their effectiveness against a wide range of viruses, including herpes simplex virus (HSV-1 and HSV-2) [37,38], influenza virus [40], hepatitis C and hepatitis E viruses [41], human immunodeficiency virus [44], and severe acute respiratory syndrome coronavirus 2 (SARS-CoV-2) [39,42,43]. The antimicrobial performance of ZnO NPs strongly depends on particle size, shape, surface area, and crystallinity, with smaller particles exhibiting higher ROS generation and stronger antimicrobial activity [46,47,48,49].

However, the tendency of ZnO NPs to aggregate may compromise their long-term stability, while uncontrolled Zn^2+^ ion release can pose toxicity risks depending on concentration and exposure routes, including dermal contact, ingestion, and inhalation [50,51]. Therefore, although ZnO NPs offer significant advantages across various industrial sectors, their widespread application remains limited due to concerns regarding their potential environmental and human health toxicity [52,53]. To address these challenges, surface modification of ZnO NPs with inorganic coatings (e.g., silica) or organic polymers such as poly(sodium 4-styrenesulfonate), poly(allylamine hydrochloride), poly(ethylene glycol), or chitin nanofibers has been employed to improve dispersion stability and biocompatibility, resulting in the formation of safer and more effective hybrid nanomaterials [54,55,56,57,58,59]. In this context, recent investigations have primarily focused on the integration of ZnO NPs with CNF to develop advanced nanohybrid systems that synergistically combine the antimicrobial functionality of ZnO with the mechanical robustness and structural versatility of cellulose [20,21]. The CNF matrix functions as a stabilizing and templating scaffold, effectively mitigating ZnO agglomeration, promoting homogeneous nanoparticle dispersion, and facilitating controlled Zn^2+^ ion release. Such synergistic interaction between the inorganic and biopolymeric components imparts reduced toxicity, prolonged physicochemical stability, and enhanced antimicrobial efficacy to the resulting nanocomposite materials. In this study, two types of bio-derived cellulose nanofibers were developed: one functionalized with carboxylate groups (anionic CNF, a-CNF) and another modified with quaternary ammonium groups (cationic CNF, c-CNF). Subsequently, two cellulose nanofibers/zinc oxide hybrid nanomaterials (a-CNF/ZnO and c-CNF/ZnO) were synthesized via a simple aqueous hydrothermal process, employing the as-prepared anionic and cationic CNF in combination with a low-cost inorganic zinc precursor. Following comprehensive physicochemical characterization, the resulting nanohybrids were systematically evaluated for their antibacterial efficacy against representative Gram-positive *Staphylococcus aureus* (*S. aureus*) and Gram-negative *Escherichia coli* (*E. coli*), as well as for their antiviral activity against bovine coronavirus and calicivirus. In addition, their toxicity was assessed on a panel of mammalian cell lines, including A549 (representative of human lung epithelium), Caco-2 (representative of human intestinal epithelium), and HaCaT (representative of human skin keratinocytes) cells, to confirm their cytocompatibility. Finally, the Life Cycle Assessment (LCA) methodology was applied as a comprehensive tool to evaluate the environmental impacts of the as-prepared cellulose nanofiber–zinc oxide hybrid nanomaterials.

## 2. Materials and Methods

### 2.1. Chemicals and Reagents

Zinc(II) nitrate hexahydrate (≥99%), NaBr, 2,2,6,6-tetramethylpiperidine-1-oxyl (TEMPO), (2,3-epoxypropyl)trimethylammonium chloride (EPTMAC), tryptic soy broth (TSB), glutaraldehyde, sodium cacodylate, poly(L-lysine), 2′,7′-dichlorodihydrofluorescein diacetate (H_2_DCFDA), and agar were purchased from Sigma-Aldrich (Poole, UK). Dulbecco’s phosphate-buffered saline (PBS), penicillin/streptomycin, trypsin/EDTA and fetal bovine serum (FBS) were obtained from Biochrom GmbH (Berlin, Germany). Thiazolyl blue tetrazolium bromide (MTT) and isopropanol were purchased from Merck KGaA (Calbiochem^®^, Darmstadt, Germany). Peptone from casein was purchased from AppliChem GmbH (Darmstadt, Germany). Luria–Bertani broth (LB) was obtained from MP Biomedicals (Illkirch, France). Viability/Cytotoxicity Assay Kit for Bacteria Live & Dead Cells was purchased from Biotium (Hayward, CA, USA). L-glutamine and antibiotics were obtained from Gibco (New York, NY, USA). Eagle’s minimum essential medium (EMEM) and sterile water were obtained from Fisher (Paisley, UK), while DMEM was purchased from VWR International (Radnor, PA, USA). Fetal calf serum (FCS) was obtained from Eurobio (Courtaboeuf, France). NaClO 6–14% solution, ethanol (technical grade), and sodium hydroxide (pure solid Supelco^®^) were bought from VWR (Part of Avantor, Leuven, Belgium). Purified eucalyptus cellulose was obtained from Ahlstrom (Malmedy, Belgium) and used as received. A549 (representative of human lung epithelium, Cat. No 300114), Caco-2 (representative of human intestinal epithelium, Cat. No 300137), and HaCaT (representative of human skin keratinocytes, Cat. No 300493) cells were purchased from Cytion GmbH (Heidelberg, Germany).

### 2.2. Preparation of Anionic (a-CNF) and Cationic (c-CNF) Cellulose Nanofibers

**Scheme 1:**
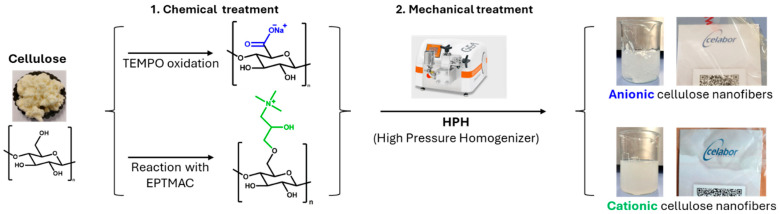
Chemical treatments of cellulose followed by mechanical defibrillation affording anionic and cationic cellulose nanofibers.

Anionic and cationic cellulose nanofibers (a-CNF and c-CNF) were produced through chemical functionalization of cellulose followed by mechanical defibrillation (Scheme 1) as described in detail below.

#### 2.2.1. Preparation of Anionic Cellulose

Cellulose (20 g) was dispersed in 1 L of deionized water using a Silverson L5M-A laboratory mixer (Silverson SARL, Evry-Courcouronnes, France). An aqueous solution (67 mL) containing 2 g of NaBr and 0.33 g of TEMPO was prepared separately and added to the dispersion, followed by the addition of 60 mL of a NaClO solution. After 10 min of reaction, the pH was adjusted to 10–11 through gradual addition of 0.5 M NaOH. The mixture was stirred at room temperature for approximately 2 h, and 1 L of ethanol was then added. The cellulose was washed with deionized water until the conductivity of the rinsing water was below 10 µS, as measured using a WTW inoLab^®^ Multi 9620 IDS meter (Xylem Analytics, Weilheim, Germany). The carboxylate-functionalized cellulose was collected by filtration and stored at 4 °C until further processing.

#### 2.2.2. Preparation of Cationic Cellulose

Cellulose (16 g) was dispersed in a mixture of 243 mL of isopropanol and 103 mL of water in a round-bottom flask and stirred at 50 °C for 15 min. A volume of 32 mL of an 8 wt. % NaOH solution was then added, followed 15 min later by the addition of 14 mL of EPTMAC. The reaction was stirred for 2 h before being quenched with 300 mL of cold water. The pH was adjusted to 6.8–7.2 using HCl, and the cellulose was washed with deionized water until the conductivity of the rinsing water fell below 10 µS (WTW inoLab^®^ Multi 9620 IDS, Xylem Analytics, Weilheim, Germany). The functionalized cellulose with quaternary ammonium groups was collected by filtration and stored at 4 °C until further processing.

#### 2.2.3. Mechanical Defibrillation to Produce a-CNF and c-CNF

Both anionic and cationic celluloses were diluted to 1 wt. % in deionized water and mechanically defibrillated using a GEA Lab High-Pressure Homogenizer PandaPLUS 2000 (GEA, Düsseldorf, Germany). Anionic cellulose underwent five homogenization passes, while cationic cellulose underwent twelve passes, yielding anionic and cationic cellulose nanofibers (a-CNF and c-CNF), respectively.

### 2.3. Preparation of a-CNF/ZnO and c-CNF/ZnO Nanohybrids Employing Cellulose Nanofibers as Templates

Dispersions of anionic or cationic cellulose nanofibers were used as templates for the synthesis of ZnO nanoparticles finely dispersed in the nanofibers. ZnO nanoparticles were in situ-prepared in cellulose nanofiber dispersions employing the hydrothermal method in alkaline medium at 70 °C. Briefly, a solution of zinc(II) nitrate hexahydrate (0.36 g in 4 mL water) was added to 30 g of 1 wt. % anionic or cationic cellulose nanofiber dispersion in water (corresponding to a Zn precursor/cellulose fiber weight ratio of 1.2) under continuous mixing, and to the resulting mixture, NaOH solution was gradually added under continuous stirring until a pH value of 12 was obtained. The resulting dispersions were kept at 70 °C for 24 h under stirring, and after reaching room temperature the dispersions were filtrated using a Büchner funnel. The resulting materials were redispersed in water and centrifuged for 3 times (10,000 rpm, 15 min) to remove any unreacted molecules. The final hybrid materials were kept as dispersions at 4 °C until used. Lyophilization was also employed in order to perform characterization experiments (FTIR and XRD). ZnO content in the dried hybrid nanohybrids was attained by heating the materials in an electric oven (STM-3-12 muffle furnace, Henan Sante Furnace Technology Co., Luoyang, China) in air up to 800 °C (heating rate 5 °C/min), which led to the complete loss of cellulose nanofibers (confirmed by performing the same procedure with parent cellulose fibers) and the determination of the residual weight. It should be noted that a number of experiments employing both lower and higher Zn precursor/cellulose fiber weight ratios from 0.5 to 2 were initially performed. Low ratios, as expected, led to very low ZnO content in the final products, while ratios higher than 1.5 lead to heavily agglomerated particles.

### 2.4. Physicochemical Characterization of Nanomaterials

Cellulose functionalization was initially confirmed by FTIR spectroscopy. Spectra were acquired using a Nicolet 6700 spectrometer (Thermo Scientific, Waltham, MA, USA) equipped with a Quest ATR diamond accessory (Specac Ltd. Orpington, Kent, UK) at 4 cm^−1^ resolution. For each spectrum, 64 scans were acquired and signal-averaged. The anionic cellulose functionalization degree was quantified by Inductively Coupled Plasma–Atomic Emission Spectrometry (ICP-AES, Optima 8300 Concentric, PerkinElmer BV, Kraainem, Belgium). After the reaction, the functionalized anionic cellulose was dispersed at 1.5 wt. % in a 4 wt. % NaCl solution to ensure that all carboxylate groups have Na^+^ as their counterion. The mixture was stirred for 1 h. The cellulose was then washed until the conductivity of the rinsing water was below 5 µS. This ensured that the excess Na^+^ (in the solution, which is not bound to cellulose) was removed before performing the ICP-AES analysis.

The cationic cellulose functionalization was quantified via conductometry titration using a WTW Inolab Multi 9620 IDS (Xylem Analytics, Weilheim, Germany). The functionalized cellulose was washed until the conductivity of the rinsing water was below 5 µS to ensure that the excess ions (in the solution, not bound to cellulose) were removed. Then, 20 mL of a 5 g/L c-CNF suspension was prepared with Milli-Q water and titrated with a 5 mM of aqueous silver nitrate solution, similarly as reported in the literature [60,61].

To verify that the employed mechanical method resulted in successful defibrillation and fiber size uniformity of a-CNF and c-CNF dispersions, prior to their use in the following experiments, we proceeded with the preparation and optical characterization of cellulose nanofibers cast films. Thus, after the defibrillation step, a film was prepared by mixing 40 g of 1 wt. % a-CNF or c-CNF dispersions with 0.4 g of pure glycerol and 25 g of water. The resulting mixtures were applied on a 10 cm Teflon mold and dried at 60 °C. The thickness of the obtained films was 40 ± 1 µm, as measured by a digital micrometer (Büchel B.V., the TMI Group of Companies, Hengelo, The Netherlands). The transmittance of the film was measured in the spectral range from 400 to 800 nm, employing a UV–vis spectrometer (GENESYS™ 150, Thermo Fisher Scientific, Inc., Madison, WI, USA). The film opacity values were calculated using the formula Opacity = −logT_600_/firm thickness, where T_600_ is the transmittance at 600 nm [62]. The viscosity of the CNF aqueous suspensions was determined by a Brookfield DV-I viscosimeter (Brookfield Engineering Laboratories, Inc.; Middleboro, MA, USA) at 50 rpm.

The *ζ*-potential values of both cellulose nanofiber as well as of the CNF/ZnO nanohybrids were measured employing a ZetaPlus instrument (Brookhaven Instruments Corp, Long Island, NY, USA). For these experiments, 0.1 wt. % aqueous dispersions of all nanofibers were employed. For each dispersion, ten measurements were collected and the results were averaged.

X-ray diffractograms of parent cellulose nanofibers and of a-CNF/ZnO and c-CNF/ZnO hybrid nanofibers after lyophilization were obtained employing a Rigaku RUH3R rotating anode generator (operating at 50 kV, 100 mA) coupled with an R-axis IV double imaging plate detector (Rigaku Co., Tokyo, Japan). The fibers were placed in Lindemann capillaries of 1 mm inner diameter. The morphology of fibers was examined by SEM (Jeol JSM 7401F Field Emission SEM, Jeol, Tokyo, Japan). Scanning transmission electron micrographs were acquired using a FEI Talos F200i field emission (scanning) transmission electron microscope (Thermo Fisher Scientific Inc., Waltham, MA, USA).

### 2.5. Assessment of Antibacterial Activity

Bacterial Strains and culture conditions: In this study, Gram (−) *Escherichia coli* strain DH5*α* (*E. coli*, NCBI Taxonomy ID: 668369) and Gram (+) *Staphylococcus aureus* strain ATCC 25,923 (*S. aureus*) were employed to evaluate the antibacterial activity of both a-CNF/ZnO and c-CNF/ZnO nanohybrids, as well as the parent anionic and cationic cellulose nanofibers, following the CLSI guidelines (documents M07-A9 and M26-A [63,64]). *E. coli* cultures were grown in Luria–Bertani (LB) medium at 35 °C under approximately 90% relative humidity for 18 h, while *S. aureus* cultures were grown in tryptic soy broth (TSB) aerobically at 37 °C for 16 h. Both bacteria strains were incubated in a Stuart SI500 orbital shaker (Bibby Scientific Ltd., Staffordshire, UK) with shaking at ~200 rpm. Bacterial inocula were adjusted to a turbidity equivalent to the 0.5 McFarland standard before use. The turbidity of *E. coli* and *S. aureus* suspensions was measured at 600 nm using a Cary 100 Conc UV–Visible spectrophotometer (Varian Inc., Mulgrave, Australia).

*Minimum Inhibitory Concentration (MIC)*: MIC values for both a-CNF/ZnO and c-CNF/ZnO hybrids, as well as for both parent cellulose nanofibers (a-CNF and c-CNF), were determined using the broth macro-dilution method, according to the CLSI M07-A9 protocol [63]. Briefly, dispersions of nanohybrids or CNF in LB or TSB media at concentrations ranging from 50 μg/mL to 1000 μg/mL were mixed with diluted bacterial subcultures (5 × 10^5^ CFU/mL). Each dispersion was mixed with an equal volume of bacterial suspension (final volume: 2 mL) and incubated for 24 h at 35 °C for *E. coli* or 37 °C for *S. aureus*. Untreated bacteria and blank media served as positive and negative controls, respectively. After incubation, the MIC was recorded as the minimal concentration where no detectable bacterial growth occurred.

*Minimum Bactericidal Concentration (MBC)*: The MBC values were determined using the colony counting method, in accordance with the CLSI M26-A protocol [64]. In brief, 100 μL aliquots were taken from the tube at MIC as well as from tubes at the higher tested concentrations. These aliquots were serially diluted and spread on agar plates. After 24 h incubation, bacterial colonies (CFU/mL) were counted, and the reduction in viable bacteria was calculated. The MBC was defined as the lowest concentration that achieved a 99.9% reduction in the initial bacterial inoculum.

*Cell viability analysis*: To quantify the effects of both CNF/ZnO nanohybrids on the bacterial populations, a Viability/Cytotoxicity Assay was used to identify the proportion of live and dead cells after incubation with nanohybrids. The fundamental principle of this assay is based on two fluorescent dyes, ethidium homodimer III (EthD-III) and 7-dimethylamino-4-methylcoumarin (DMAO), which distinguish between live and dead cell populations. Specifically, EthD-III is a red fluorescent nucleic acid dye that stains only cells with compromised membranes (dead or dying cells), while DMAO is a bright green fluorescent nucleic acid dye that stains both live and dead bacteria. In this assay, 1 × 10^8^ CFU/mL bacterial suspensions were treated with a-CNF/ZnO and c-CNF/ZnO hybrids at various concentrations ranging from 50 μg/mL to 400 μg/mL. The mixtures were incubated at 37 °C in a Stuart SI500 orbital shaker (~200 rpm) for 2 and 5 h. Untreated bacteria served as the positive control (live and dead cells), while the bacteria obtained after heating to 90 °C for 5 min and then allowing to cool, served as the negative control (dead cells). After the incubation time, samples were pelleted by centrifugation at 10,000× *g* for 5 min and resuspended in 100 μL of 150 mM NaCl. Subsequently, 1 μL of dye mixture (1 μL DMAO, 2 μL EthD-III and 8 μL 150 mM NaCl) was added to each sample, followed by incubation at room temperature for 15 min in the dark. The fluorescence intensity was measured (EthD-III: λ_ex_ = 532 nm; λ_em_ = 625 nm, DMAO: λ_ex_ = 497 nm; λ_em_ = 528 nm) using an Infinite M200 microplate reader (Tecan Group Ltd., Männedorf, Switzerland). Data were expressed as the percentage of dead cells, calculated from the ratio of EthD-III fluorescence intensity (dead cells) to DMAO fluorescence intensity (total cells) (mean ± SD, *n* = 3). Each concentration was tested in eight replicates, and all experiments were performed in triplicate. Statistical significance was calculated by comparing the a-CNF/ZnO and c-CNF/ZnO hybrid datasets using a paired two-tailed Student’s *t*-test. Statistical significance is denoted as * *p* < 0.05, *** *p* < 0.001, **** *p* < 0.0001; absence of annotation indicates no significance (*p* > 0.05).

*Intracellular Reactive Oxygen Species (ROS) production*: To assess the intracellular ROS levels, the 2′,7′-dichlorodihydrofluorescein diacetate (H_2_DCFDA) assay was used. H_2_DCFDA is a membrane-permeable dye that is deacetylated by intracellular esterases to the non-fluorescent 2′,7′-dichlorodihydrofluorescein (DCFH), which is then oxidized by ROS to form the highly fluorescent 2′,7′-dichlorofluorescein (DCF) [65]. Specifically, bacterial suspensions (1 × 10^8^ CFU/mL) were treated with a-CNF/ZnO and c-CNF/ZnO hybrids at various concentrations ranging from 50 μg/mL to 300 μg/mL. The mixtures were incubated at 37 °C in a Stuart SI500 orbital shaker (~200 rpm) for 2 and 5 h. Untreated bacteria served as controls. After the incubation time, 10 μM H_2_DCFDA was added to each sample, followed by incubation in the dark at 30 °C for 45 min. The suspensions were then centrifuged, washed, resuspended in PBS, ultrasonicated to disrupt cells, and centrifuged again. The fluorescence intensity of the supernatant was measured (λ_ex_ = 485 nm; λ_em_ = 530 nm) using an Infinite M200 microplate reader (Tecan Group Ltd., Männedorf, Switzerland). Each concentration was tested in six replicates, and all experiments were performed in triplicate. Statistical significance was calculated by comparing the a-CNF/ZnO and c-CNF/ZnO hybrid datasets using a paired two-tailed Student’s *t*-test. Statistical significance is denoted as ** *p* < 0.01, *** *p* < 0.001, **** *p* < 0.0001.

*Bacterial Morphological Analysis*: The morphological changes in *S. aureus* bacteria after treatment with a-CNF/ZnO and c-CNF/ZnO hybrids were examined by SEM (Jeol JSM 7401F Field Emission SEM, Jeol, Tokyo, Japan). Cells were exposed to each hybrid at a concentration corresponding to ½MIC for 24 h. Treated *S. aureus* cells were fixed with 3 wt. % glutaraldehyde in 100 mM sodium cacodylate buffer (pH = 7.1) for 12 h, then washed to remove excess fixative and by-products. The cells were resuspended in the same buffer, and 50 μL of each suspension was placed on poly(L-lysine)-coated glass coverslips. The samples were dehydrated through a graded ethanol series (50%, 70%, 95%, and 100%) for 10 min each, air-dried, and finally gold-coated using a sputter coater before SEM imaging [66,67].

### 2.6. Assessment of Antiviral Activity

Viral Strains, permissive cells and culture conditions: In this study, enveloped bovine coronavirus (BCoV, FLI RVB-0020) and non-enveloped feline calicivirus (FCV, FLI RVB-0208) were obtained from Friedrich-Loeffler-Institut (FLI), Insel Riems, Germany and employed to evaluate the antiviral activity. All viruses were produced on specific cell lines: Bovine kidney epithelial MDBK (FLI CCLV-RIE261) for BCoV and Crandell-Rees Feline Kidney CRFK (FLI CCLV-RIE0138) for FCV, which were obtained from the Collection of Cell Lines in Veterinary Medicine (CCLV) of Friedrich-Loeffler-Institut (FLI). Cells were cultivated in EMEM supplemented with L-glutamine, antibiotics, and a controlled quantity of fetal calf serum (FCS, 2%) for BCoV. Viral cultures were grown in EMEM at 37 °C and 5% CO_2_ for 48 to 72 h. Viral titers were determined using the 50% tissue culture infectious dose (TCID_50_) in cell culture microtitre plates and calculated using the Spearman–Kärber quantal method.

*Antiviral activity analysis*: Antiviral activity was assessed according to an adapted NF EN 14476 (2019) standard protocol [68]. A 10 mL volume of the product, diluted with sterile water, was added to 10 mL of the viral test suspension. The mixture was maintained at 20 ± 1 °C for 24 h of contact time under mechanical agitation. At the end of the contact time, an aliquot was taken, and the virucidal action in this portion was immediately suppressed by a 1/10 dilution in ice-cold cell maintenance medium. Tenfold serial dilutions were transferred into cell culture microtitre plates (Sarstedt, Nümbrecht, Germany) containing monolayers of permissive cells. After 5 and 6 days of incubation for BCoV and FCV, respectively, at 37 °C with 5% CO_2_, infectivity titers were calculated according to the Spearman–Kärber quantal method (TCID_50_/mL). The reduction (R) in viral infectivity was calculated from differences in log_10_ viral titers before (virus control) and after treatment with the product.

To check the potential cytotoxicity (morphological alteration of cells) caused by the product test solutions, 2 parts of sterile water were mixed with 8 parts of the product test solution. Serial dilutions were prepared in culture medium and inoculated onto cell monolayers. Any microscopic changes in the cells were recorded, and the cytotoxicity level was determined using the Spearman–Kärber quantal method.

### 2.7. In Vitro Assessment of Cytotoxicity

The cytotoxicity of both a-CNF/ZnO and c-CNF/ZnO hybrids, as well as for both parent cellulose nanofibers (a-CNF and c-CNF), was assessed using the standard MTT assay. A549 (representative of human lung epithelium), Caco-2 (representative of human intestinal epithelium), and HaCaT (representative of human skin keratinocytes) cells were cultured in Dulbecco’s Modified Eagle Medium (DMEM) supplemented with 10% (*v*/*v*) fetal bovine serum (FBS), 1% (*v*/*v*) non-essential amino acid mixture, 2 mM L-glutamine, 100 μg/mL streptomycin, and 100 U/mL penicillin. Cells were maintained at 37 °C in a humidified atmosphere containing 5% CO_2_. For the MTT assay, when cell confluence exceeded 70%, cells were detached using trypsin, seeded into 96-well plates at a density of 7.5 × 10^3^ cells per well and incubated in complete medium for 24 h. After this pre-incubation period, cells were treated with various concentrations of the tested materials (6.25–100 μg/mL) for 24 h. Subsequently, an MTT stock solution (5 mg/mL in sterile PBS) was prepared, protected from light due to photosensitivity, and filtered through a 0.22 μm membrane (Millipore^®^, Darmstadt, Germany). The culture medium was removed, and cells were washed with PBS before adding 100 μL of DMEM containing 20% of the MTT stock solution. Plates were incubated for 3 h to allow viable cells to reduce MTT into insoluble formazan crystals. The supernatant was then discarded, and the formazan crystals were dissolved in dimethyl sulfoxide. The resulting violet coloration was quantified spectrophotometrically by measuring absorbance at 570 nm with a reference wavelength of 620 nm using a Thermo Scientific^TM^ Multiskan^TM^ FC 96-well microplate photometer.

Cell viability (%) was calculated relative to untreated controls (cells incubated in complete medium). Each concentration was tested in sixteen technical replicates per 96-well plate, and all experiments were performed in triplicate. Statistical analysis was performed using one-way ANOVA followed by Tukey’s multiple comparison test, with differences considered significant at *p* ≤ 0.05, indicated as *; absence of annotation indicates no significance (*p* > 0.05).

### 2.8. LCA of CNF/ZnO Nanohybrids

Life Cycle Assessment (LCA) provides an integrated framework to evaluate the environmental potential impacts associated with a product throughout its entire lifespan, from raw material extraction to end-of-life management. The procedure followed the principles and requirements outlined in ISO 14040 and ISO 14044 [69,70], which define four main stages: defining the goal and scope, compiling the life cycle inventory (LCI), conducting the life cycle impact assessment (LCIA), and interpreting the results.

#### 2.8.1. Goal and Scope

In this study, the LCA aims to quantify and compare the cradle-to-gate environmental impacts of two CNF/ZnO nanohybrids derived from anionic and cationic cellulose nanofibers. Both nanohybrids, a-CNF/ZnO and c-CNF/ZnO, follow a comparable synthesis pathway.

#### 2.8.2. Functional Unit and System Boundaries

The evaluation encompasses the extraction and processing of raw materials, chemical reagents, and energy inputs, as well as the synthesis of the CNF/ZnO hybrids under the two respective modification pathways. The analysis excludes the use and end-of-life stages, thereby defining a cradle-to-gate system boundary. The functional unit is specified as 1 g of CNF/ZnO nanohybrid, providing a mass-based reference for a direct comparison between the two synthesis routes, independent of their potential applications.

**Figure 1:**
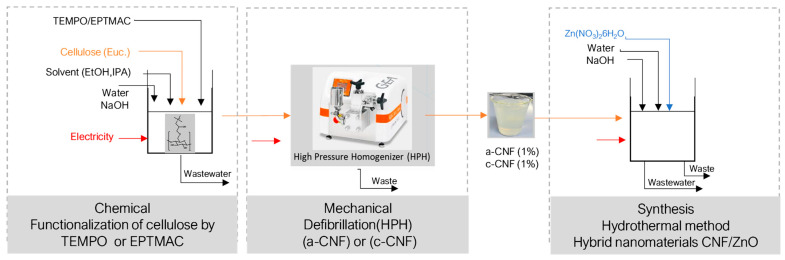
System boundaries for the evaluation of CNF/ZnO nanohybrids at the laboratory scale.

The system boundaries (Figure 1) were defined to include all major processes contributing to the product system, covering the material and energy flows across the life cycle stages considered: (i) chemical functionalization of cellulose using TEMPO to obtain the anionic precursor or EPTMAC to obtain the cationic precursor, (ii) high-pressure homogenization for mechanical defibrillation to produce anionic (a-CNF) or cationic (c-CNF) cellulose nanofibers, and (iii) hydrothermal synthesis to obtain CNF/ZnO hybrid nanomaterials.

#### 2.8.3. Life Cycle Inventory

The life cycle inventory (LCI) was primarily based on data collected directly from laboratory-scale (Table S1) experiments conducted as part of this study. Environmental impacts were then quantified using the adapted EF 3.1 as recommended by the European Commission (Commission Recommendations 2013/179/EU) [71], implemented through SimaPro software (version 10.2.0.1) together with the Ecoinvent v3.11 database.

## 3. Results and Discussion

### 3.1. Synthesis and Physicochemical Characterization of Functionalized Cellulose Nanofibers

#### 3.1.1. Anionic Cellulose Nanofibers (a-CNF)

The reaction of the cellulose with TEMPO led to the oxidation of the cellulose. This method converts the primary alcohol groups of cellulose into carboxylate groups with Na^+^ as the counterion (Scheme 1) [72]. As is known in the literature, the degree of functionalization of cellulose with TEMPO will directly influence its properties [73]. The ICP-AES analysis allowed the determination the Na content (g) per gram of a-CNF. Then, the –COONa equivalent is calculated using its molar mass, and finally the mol. % of substitution can be derived. In the present sample, the a-cellulose was functionalized with 20 mol. % of carboxylate groups. The presence of the carboxylate groups is also manifested in the electrophoretic mobility of their dispersions. The *ζ*-potential values obtained at neutral pH were found to be −55.6 ± 0.3 mV, confirming the presence of carboxylates and pointing to stable dispersions due to electrostatic repulsion between the nanofibers.

**Figure 2:**
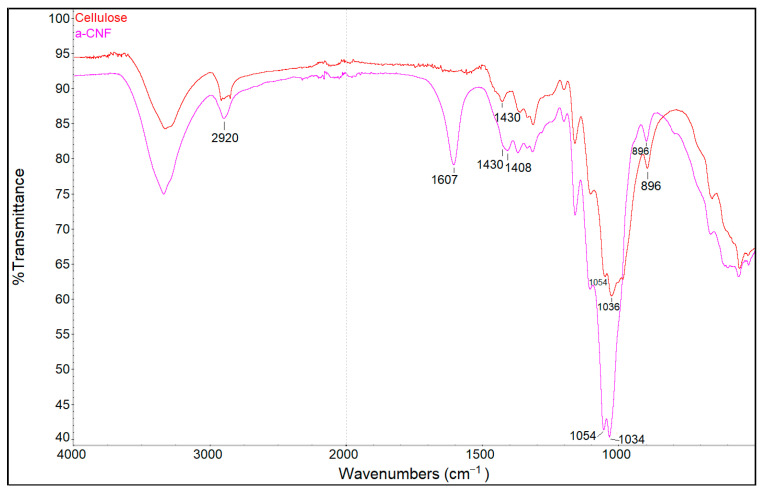
FTIR spectra of purified cellulose and anionic cellulose nanofibers (a-CNF).

Infrared spectroscopy was also employed to confirm the grafting of carboxylate groups. The FTIR spectrum of a-CNF (Figure 2) has all the characteristic infrared bands of cellulose, including the stretching bands of hydrogen-bonded OH groups in the 3100–3600 cm^−1^ region, the symmetric C–H vibration band at 2920 cm^−1^, the band at 1430 cm^−1^ assigned to a symmetric CH_2_ bending vibration, and the most intense bands of the spectrum at 1054 cm^−1^ and at 1034 cm^−1^ both attributed to the C–O stretching and C–O–C stretching skeletal vibrations of glycosidic linkages in cellulose (C–O–C) and in xylose-containing hemicellulose, respectively, as well as the sharp band at 896 cm^−1^ assigned to C1–H bending of both cellulose and xylose-containing hemicelluloses (Figure 2) [74,75,76,77,78]. In addition, two new prominent bands in the spectrum of a-CNF are located at 1607 cm^−1^ and at 1408 cm^−1^ attributed to the anti-symmetric and symmetric stretching vibrations of the ionized carboxylate groups (COO^−^), respectively [79], attesting the presence of carboxylate moieties in anionic nanofibers.

The high-pressure homogenization (HPH) process applies shear forces to the cellulose fibers, leading to their shortening and thinning to the nanofiber scale. The degree of micro- and nano-fibrillation of the resulting cellulose depends on the number of cycles through the machine, the applied pressure, and the material properties (cellulose purity, type of functionalization, degree of functionalization, etc.) [80]. Following the defibrillation step, the viscosity of the obtained a-CNF suspension (1 wt. % aqueous gel) was registered at 50 rpm. The obtained value was high (3050 cP), indicating a high degree of defibrillation of cellulose [81]. In addition, an a-CNF film was prepared and its UV–vis spectrum was acquired to provide additional information on the quality of defibrillation [81], as it is well-established that cellulose film transparency is considered a quality index of cellulose nanofibrils [81]. As in our previous work on chitin nanofibers [59], the transmission of the a-CNF film having a thickness of 40 μm was between 75 and 85% in the whole 400–800 nm region (Figure S1), which confirmed the high efficiency of the defibrillation step and indicate the presence of thin and short fibers [81]. The transmittance at 600 nm is 76% that results in a calculated opacity value of 3.

**Figure 3:**
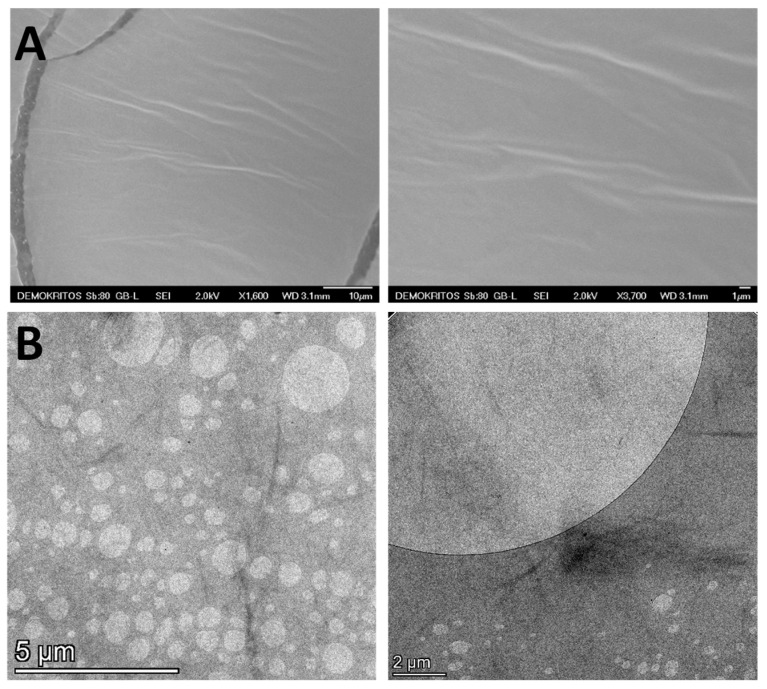
SEM (**A**) and TEM (**B**) micrographs of a-CNF.

The morphology of a-CNF fibers was also investigated using SEM and TEM. As shown in the SEM micrographs (Figure 3A), most of the nanofibers are observed to be de-bundled and no aggregation of nanofibers is detected. TEM images, at much higher magnifications (Figure 3B), reveal the presence of a few loosely bound bundles of nanofibers of ~2 to 5 μm in length and 100–200 nm in thickness, while most of the unbundled nanofibers have widths of <50 nm.

#### 3.1.2. Cationic Cellulose Nanofibers (c-CNF)

As reported in the literature [82], the reaction between the primary alcohol of cellulose with EPTMAC yields cationized cellulose bearing quaternary ammonium groups (Scheme 1). The extent of grafting was quantified by conductometric titration with AgNO_3_ [60,61]. In the present study, the equivalence point was identified at 8.6 mL of AgNO_3_, as shown in Figure S2. The degree of functionalization was subsequently determined from the molar ratio of [AgNO_3_]/[cellulose], resulting in a value of 7.75 mol %.

The presence of the quaternary ammonium groups was also evident in the electrophoretic mobility of c-CNFs. Their *ζ*-potential values obtained at neutral pH were found to be +45.1 ± 0.6 mV, confirming the observed aqueous stability of their dispersions due to the strong electrostatic repulsion between the nanofibers.

**Figure 4:**
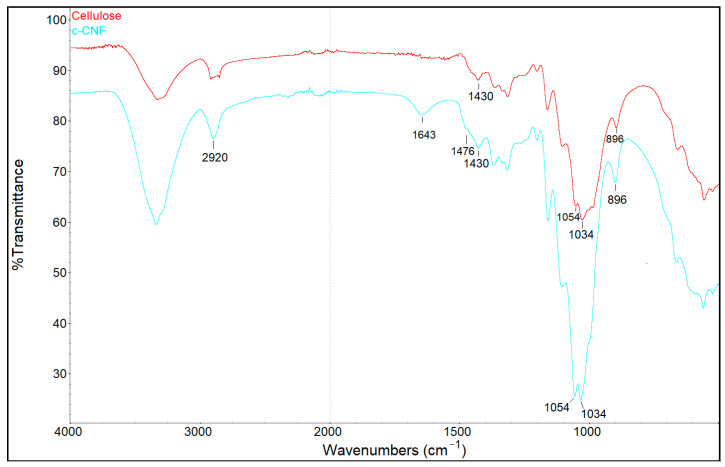
FTIR spectra of purified cellulose and cationic cellulose nanofibers (c-CNF).

The successful introduction of quaternary ammonium groups into cellulose was confirmed by infrared spectroscopy. Specifically, the FTIR spectrum of c-CNF (Figure 4) retains all characteristic bands of parent cellulose as quoted above, and additionally displays a new band at 1476 cm^−1^ attributed to the CH_3_–N^+^ stretching vibrations of the quaternary ammonium group [61]. The band at 1643 cm^−1^ corresponds to absorbed water, consistent with the enhanced hydrophilicity imparted by the quaternary ammonium groups [75,83]. Additionally, due to the formation of new ether linkages, an increase in the intensity of the band at 1054 cm^−1^, attributed to the ether C–O– stretching vibration [79], is also observed.

Following the defibrillation step as described above, the viscosity of the resulting c-CNF dispersion (1 wt. %) at 50 rpm was 3400 cP, indicating a high degree of defibrillation of the cellulose, as explained previously for the a-CNF. The light transmission of a film of c-CNF with a thickness of 40 μm was between 63 and 73% in the whole 400–800 nm region (Figure S1). The transmittance at 600 nm is 72% and the calculated opacity value is ca. 3.5, i.e., slightly less transparent than that of a-CNF. This observation coupled with a viscosity which is also higher than that of the a-CNF dispersion, suggest that the c-CNF are, to some degree, more difficult to defibrillate.

**Figure 5:**
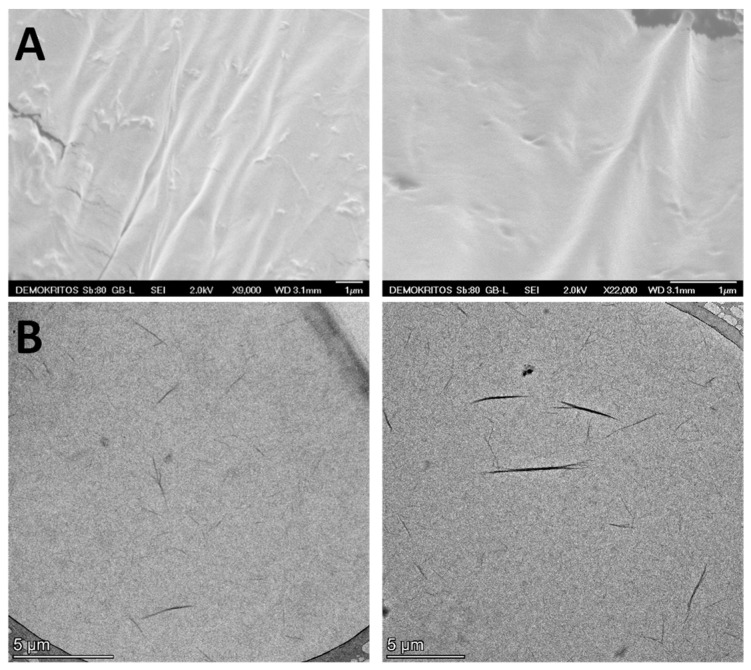
SEM (**A**) and TEM (**B**) micrographs of c-CNF.

The morphology of cationic cellulose nanofibers was also studied using SEM and TEM as above. As shown in the SEM micrographs (Figure 5A), most of the nanofibers are observed to be de-bundled, but some aggregations of nanofibers are also detected. Higher-magnification TEM images (Figure 5B) show that the present bundled nanofibers have the same length as in a-CNF (~2 to 5 μm) but are considerably less thick (2–5 nm) and denser, which is in line with the viscosity and absorbance spectroscopy results that both suggest a less efficient defibrillation.

### 3.2. Synthesis and Physicochemical Characterization of a-CNF/ZnO and a-CNF/ZnO Nanohybrids

The hydrothermal method employed for the preparation of nanostructured materials is widely employed and several reviews on this method highlight its simplicity and environmental friendliness [84,85]. In this study, it was attested that cellulose nanofibers act as templates leading to the formation of ZnO nanoparticles at relatively low temperatures (70 °C) in water without the use of any additional solvents or chemicals. The *ζ*-potential values in water were found to be −34.2 ± 1.1 mV for the negatively charged a-CNF/ZnO and +38.2 ± 1.4 mV for the positively charged c-CNF/ZnO nanohybrid. For both cases, the obtained values point to stable dispersions. Lyophilized nanohybrids were employed for further characterization by TEM, FTIR, and XRD. In addition, after heating the lyophilized compounds at 800 °C and determining the weight loss, it was found that the ZnO content was 30 ± 1 wt. % in a-CNF/ZnO and 20 ± 1 wt. % in c-CNF/ZnO nanofibers.

**Figure 6:**
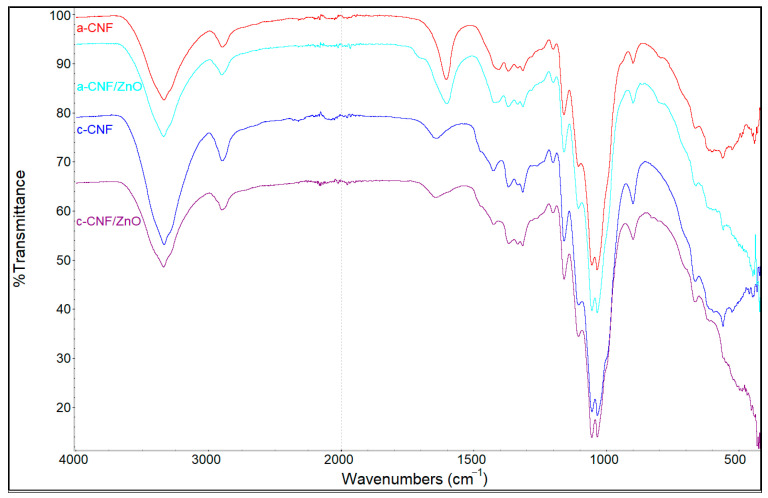
FTIR spectra of anionic (a-CNF) and cationic (c-CNF) cellulose nanofibers, as well as of the corresponding anionic and cationic cellulose/ZnO nanohybrids (a-CNF/ZnO and c-CNF/ZnO, respectively).

The bands in the FTIR spectra of both anionic and cationic cellulose fibers in the 3600–3100 cm^−1^ region are attributed to the OH-stretching modes and are rather sharp and centered at 3238 cm^−1^ (Figure 6). This suggests the presence of amorphous regions that are known to result in sharp vibration modes in this region due to the scission of the intramolecular and intermolecular hydrogen bonds [77]. On the other hand, the position of the band at ca. 2900 cm^−1^ attributed to the C-H stretching vibration points to crystalline cellulose content. In addition, the presence in both samples of a band at 1430 cm^−1^, known as the “crystallinity band” which is assigned to the symmetric bending of CH_2_, also indicate the presence of cellulose crystallites. Overall, FTIR spectra designate that both amorphous and crystalline areas are present in the CNFs. The spectra of the corresponding CNF/ZnO nanohybrids are essentially identical to those of the original nanofibers, indicating that the employed hydrothermal procedure for ZnO formation does not affect the physical state of the parent cellulose fibers. Indeed, the FTIR spectra of the hybrid ZnO/cellulose nanofibers exhibit all the peaks of the parent cellulose nanofibers in the 4000–600 cm^−1^ region. The observed strong absorption below ca. 600 cm^−1^ for both a-CNF/ZnO and c-CNF/ZnO is indicative of the presence of ZnO nanoparticles as it is well-established that the Zn–O stretching modes of ZnO nanoparticles appear as broad bands normally centered in the 420–410 cm^−1^ region [86,87,88].

**Figure 7:**
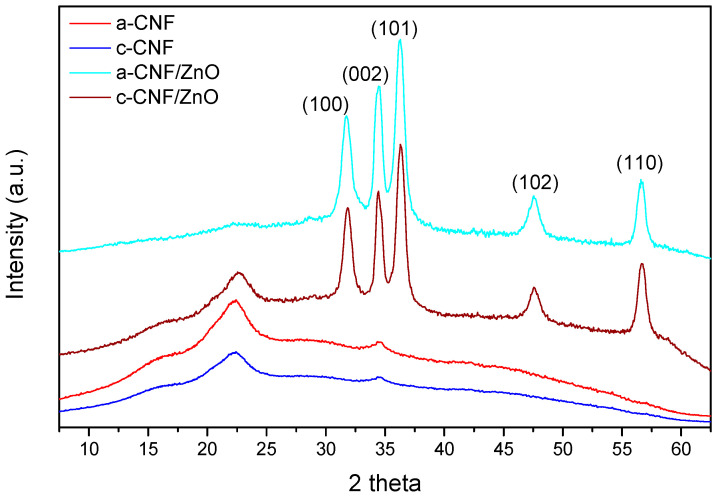
XRD patterns of anionic (a-CNF) and cationic (c-CNF) cellulose nanofibers, as well as of the corresponding anionic and cationic cellulose/ZnO nanohybrids (a-CNF/ZnO and c-CNF/ZnO, respectively).

The X-ray patterns of both the parent polymeric fibers and of the hybrid cellulose/ZnO nanofibers after lyophilization are shown in Figure 7. For both the anionic and cationic cellulose fibers, the characteristic diffraction peaks (both anionic and cationic fibers have the same XRD profile after lyophilization) suggest that they are amorphous, containing also cellulose I crystallites. The most intense peak of the nanofibers centered at 22.4 degrees can be assigned to the (110) or the (200) peak of the Iα or Ιβ crystallites, respectively, while the peak at 34.5 degrees can be assigned to either the (210) or the (004) peak of the Iα or Ιβ crystallites, respectively [89]. In the diffractograms of a-CNF/ZnO and c-CNF/ZnO nanohybrids, in addition to the peaks allocated to cellulose crystallites, the peaks characteristic of ZnO crystals are clearly observed. Specifically, the strong diffraction peaks with 2 theta values of 31.8, 34.5, 36.3, 47.5 and 56.7 correspond to the crystal planes (100), (002), (101), (102) and (110), respectively, of the hexagonal wurtzite structure of zinc oxide (JCPDS Card No: 36-1451). Employing the Debye-Scherrer equation and assuming that the line broadening was caused entirely by particle size, for the Gaussian fitted diffraction peak of (100) plane, the crystallite size of ZnO in cationic cellulose fibers was found to be 15.7 nm, while the corresponding size derived from the peak of the (002) plane was 21.6 nm, suggesting that the crystallites are slightly elongated along the c-axis. In the case of ZnO in anionic cellulose fibers, the respective values are similar (14.6 nm and 19.1 nm along the a and c axis, respectively), indicating that in both samples the ZnO crystallites derived employing the hydrothermal treatment are quite similar and possibly slightly elongated which can be attributed to the presence of nanofibers acting as templates during their synthesis.

**Figure 8:**
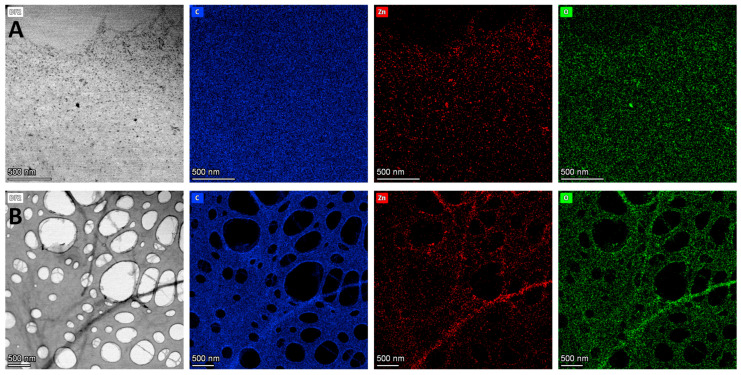
Scanning transmission electron microscopy (STEM) images of a-CNF/ZnO (**A**) and of c-CNF/ZnO (**B**) nanohybrids and the corresponding EDX elemental mapping images: C (blue), Zn (red) and oxygen (green); scale bar length is 500 nm.

The morphology of a-CNF/ZnO and of c-CNF/ZnO nanohybrids was investigated by TEM microscopy. In the case of a-CNF/ZnO, nanofibers are shown to be well-defibrillated, while ZnO nanoparticles are well-dispersed within the polymeric matrix as confirmed by the corresponding energy-dispersive X-ray (EDX) mapping images (Figure 8A). Their sizes are found to range between ca. 10–15 nm, but aggregates of about 20–30 nm can also be detected. On the other hand, STEM images of c-CNF/ZnO nanohybrid clearly show the presence of both defibrillated nanofibers and also of bundled nanofibers as also observed in TEM images of the original c-CNFs (Figure 5B). ZnO nanoparticles are located both on defibrillated nanofibers as well as on the bundled nanofibers as illustrated in the corresponding energy-dispersive X-ray (EDX) mapping images (Figure 8B). Their size is comparable with the respective sizes in a-CNF/ZnO, ranging between 10 and 15 nm.

### 3.3. Evaluation of Antibacterial Activity

The antibacterial activity of both cellulose nanofiber–ZnO nanohybrids as well as the parent anionic and cationic cellulose nanofibers was evaluated against Gram (−) *E. coli* and Gram (+) *S. aureus* bacteria by determining the MIC and MBC values, in accordance with the CLSI guidelines (documents M07-A9 and M26-A, respectively) [63,64]. The obtained results are summarized in Table 1. The parent anionic and cationic cellulose nanofibers (a-CNF and c-CNF) exhibited no detectable antibacterial activity, with MIC and MBC values higher than 500 µg/mL for both bacterial strains. These results confirm the inert nature of the cellulose nanofibers toward bacterial growth, which is in line with the literature [90]. In contrast, both cellulose nanofiber/ZnO nanohybrids exhibited significantly enhanced antibacterial performance, with the c-CNF/ZnO being more active than a-CNF/ZnO. Specifically, a-CNF/ZnO demonstrated higher activity against *S. aureus*, with MIC and MBC values of 100 and 300 µg/mL, respectively, than against *E. coli* (MIC/MBC values higher than 500 µg/mL). On the other hand, the c-CNF/ZnO hybrid showed superior antibacterial efficacy, with MIC and MBC values of 200 and 300 µg/mL for *E. coli* and of 50 and 100 µg/mL for *S. aureus*, respectively. These results clearly demonstrate that both nanohybrids were more active against *S. aureus* than against *E. coli*, in agreement with previous observations that Gram-positive bacteria are generally more sensitive to ZnO-based nanomaterials [48,59], due to differences in cell wall composition and permeability [91]. Specifically, Gram-positive bacteria possess a thick but more permeable peptidoglycan layer, which allows easier access of nanoparticles and released Zn^2+^ ions to the cell membrane. In contrast, Gram-negative bacteria have an outer membrane enriched with lipopolysaccharides that acts as a physical and electrostatic barrier, reducing the interaction and penetration of ZnO species. These intrinsic structural differences likely contribute to the higher MIC/MBC values observed for *E. coli*.

**Figure 9:**
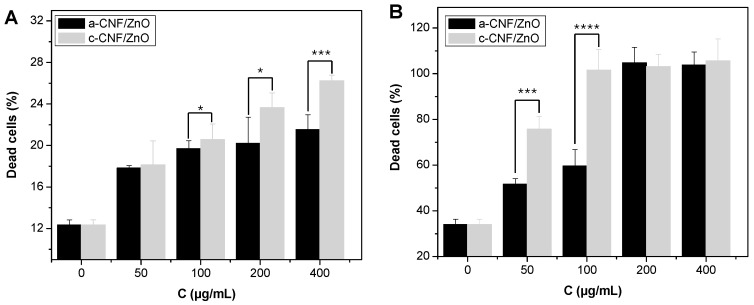
Impact of CNF/ZnO nanohybrids on bacterial viability assessed using Live/Dead staining assays. *S. aureus* cells were exposed to CNF/ZnO nanohybrids (50–400 µg/mL) at 37 °C for 2 h (**A**) and 5 h (**B**). Data are expressed as the percentage of dead cells, calculated from the ratio of EthD-III fluorescence intensity (dead cells) to DMAO fluorescence intensity (total cells) (mean ± SD, *n* = 3). Statistical significance of differences between a-CNF/ZnO and c-CNF/ZnO hybrids (Student’s paired two-tailed *t*-test) is indicated as * *p* < 0.05, *** *p* < 0.001, and **** *p* < 0.0001; absence of annotation indicates no significance (*p* > 0.05).

As both nanohybrids exhibited enhanced activity on *S. aureus* bacteria, further studies were conducted only on *S. aureus* bacteria. Thus, the impact of the nanohybrids on bacterial viability was further assessed by Live/Dead staining assays (Figure 9). Both nanohybrids induced a concentration-dependent increase in the proportion of dead cells after 2 and 5 h of exposure. Consistent with the MIC/MBC results, c-CNF/ZnO displayed significantly higher bactericidal activity than a-CNF/ZnO across the full concentration range examined. After 5 h, treatment with c-CNF/ZnO at concentrations higher than 100 μg/mL resulted in a near-complete loss of viability, whereas a-CNF/ZnO required higher concentrations to achieve comparable effects (>200 μg/mL). These findings confirm the superior antibacterial performance of the c-CNF/ZnO nanohybrid and indicate that surface charge plays a critical role in enhancing bactericidal interactions. Electrostatic attraction between the positively charged nanohybrid and the negatively charged bacterial cell wall promotes intimate contact, facilitating ZnO interaction and ion release.

It is well established that the antibacterial mechanism of ZnO NP action is related to strong electrostatic interactions with bacterial membranes, ROS production, and the release of Zn^2+^ ions [33,34,35,36]. Specifically, the antibacterial action of ZnO NPs begins with their electrostatically driven attachment to the bacterial membrane, followed by membrane disruption and eventual cell death. Additionally, the wide band gap of ZnO (~3.3 eV) facilitates the formation of ROS, including hydroxyl radicals and hydrogen peroxide, under light or even dark conditions, resulting in oxidative damage to bacterial membranes and intracellular components [34,35]. Furthermore, the Zn^2+^ ions released disrupts cell wall integrity and interferes with essential enzymatic processes, enhancing antibacterial efficacy. In this study, to probe the mechanisms responsible for the antibacterial activity of the nanohybrids, two complementary analyses were conducted: measurement of intracellular ROS production and examination of bacterial morphology after treatment.

**Figure 10:**
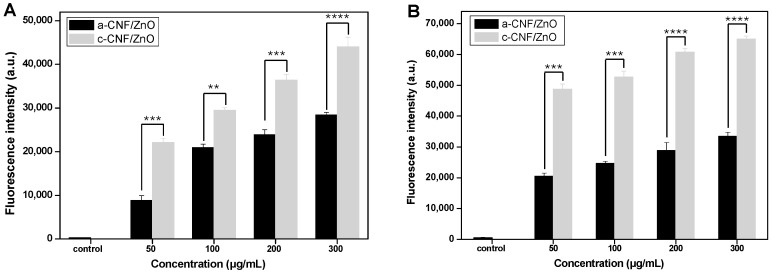
Intracellular reactive oxygen species production in *S. aureus* following treatment with a-CNF/ZnO or c-CNF/ZnO nanohybrids. Bacteria were incubated with nanohybrids at concentrations of 50–300 μg/mL for 2 h (**A**) and 5 h (**B**) at 37 °C. The fluorescence intensity of the produced 2′,7′-dichlorofluorescein (DCF) was measured (λ_ex_ = 485 nm; λ_em_ = 530 nm). Untreated cells served as the control. Each concentration was tested in eight replicates, and all experiments were performed in triplicate. Statistical significance of differences between a-CNF/ZnO and c-CNF/ZnO hybrids (Student’s paired two-tailed *t*-test) is indicated as ** *p* < 0.01, *** *p* < 0.001, **** *p* < 0.0001.

Oxidative stress is a key antibacterial mechanism of many nanomaterials, as the generated reactive oxygen species (ROS), including superoxide anions (O_2_^−^), hydrogen peroxide (H_2_O_2_), and hydroxyl radicals (•OH), can damage bacterial membranes and essential biomolecules [92]. To evaluate ROS formation induced by the nanohybrids, intracellular ROS levels were measured using the H_2_DCFDA assay. After entering bacterial cells, H_2_DCFDA is enzymatically converted to non-fluorescent DCFH, which is subsequently oxidized by ROS to form fluorescent DCF. Bacteria treated with different concentrations of a-CNF/ZnO or c-CNF/ZnO were stained with H_2_DCFDA, and the resulting DCF fluorescence was recorded at 530 nm as an indicator of intracellular ROS production. Both a-CNF/ZnO and c-CNF/ZnO induced a clear increase in ROS levels in a concentration- and time-dependent manner, with markedly higher fluorescence intensities observed after 5 h of exposure (Figure 10). In line with the viability data and MIC/MBC results, c-CNF/ZnO generated substantially higher ROS levels than a-CNF/ZnO at equivalent concentrations. Since ROS accumulation is known to contribute to oxidative stress, membrane disruption, and cellular damage, these results suggest that ROS generation is a key mechanism underlying the enhanced antibacterial activity of the nanohybrids, particularly the cationic one [33,34,35,36]. The higher ROS induction by c-CNF/ZnO correlates with its superior antibacterial activity, indicating that enhanced nanoparticle–cell interaction mediated by surface charge directly influences the extent of oxidative stress.

**Figure 11:**
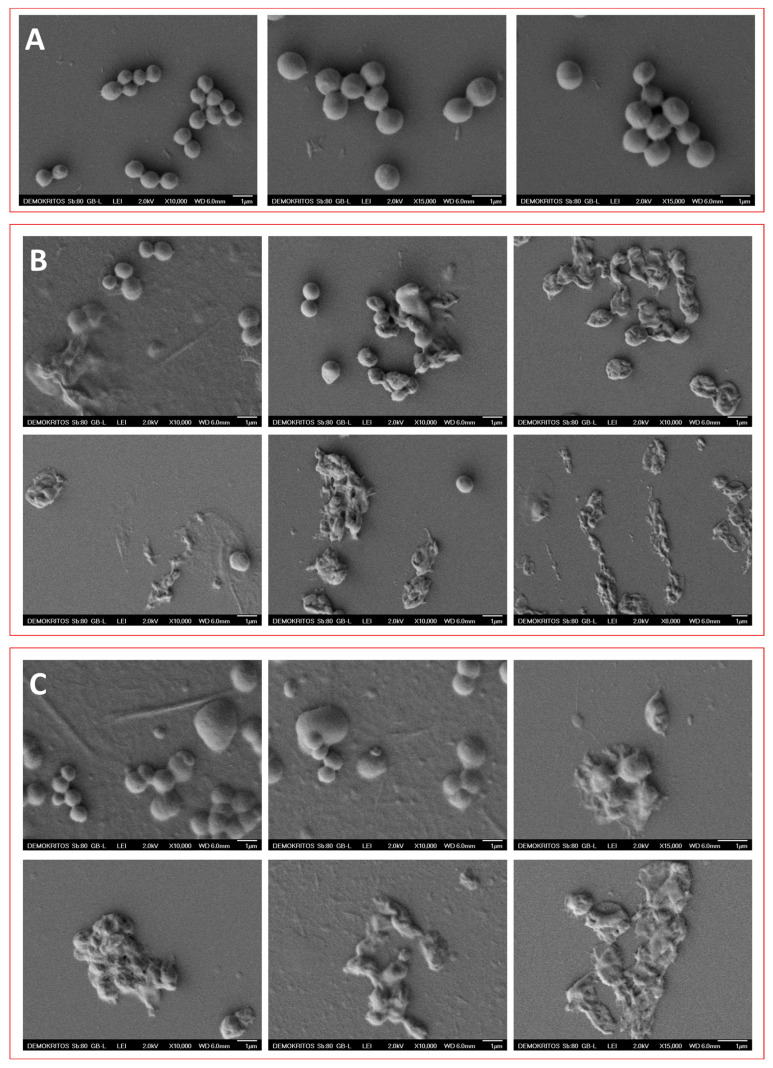
SEM images of *S. aureus* bacteria: untreated cells (**A**) as well as treated cells with a-CNF/ZnO (**B**) and c-CNF/ZnO (**C**) at 1/2 MIC for 12 h at 37 °C.

Furthermore, the morphological changes induced in *S. aureus* bacteria after treatment with a-CNF/ZnO or c-CNF/ZnO at ½MIC was investigated by scanning electron microscopy (SEM). Untreated cells exhibited the expected smooth, spherical morphology with intact surfaces (Figure 11A). In contrast, cells treated with either a-CNF/ZnO (Figure 11B) or c-CNF/ZnO (Figure 11C) displayed notable structural alterations, including surface roughening, membrane deformation, and partial collapse. In addition, the leakage of intracellular contents and changes in the shape of the cells are evident, suggesting loss of cell viability. The observed morphological alterations are characteristic effects of ROS-mediated oxidative damage combined with physical interactions between nanoparticles and the bacterial envelope.

Taken together, the results demonstrate that CNF/ZnO nanohybrids exhibit potent antibacterial activity driven by a multi-step mechanism that acts synergistically to kill the bacteria, and involves (a) electrostatic interaction between cationic surfaces and negatively charged bacterial envelopes, enhancing adhesion and nanoparticle accumulation; (b) ZnO-mediated ROS generation, leading to oxidative stress and biomolecular damage; and (c) membrane disruption, confirmed by SEM, ultimately resulting in cell death. The c-CNF/ZnO hybrid outperforms the a-CNF/ZnO system across all assays due to its cationic surface, which enhances bacterial binding and amplifies both ROS production and membrane damage. These findings underscore the importance of surface charge engineering in designing effective antibacterial nanomaterials.

Our findings are consistent with recently reported ZnO–biopolymer antibacterial systems. Topçu et al. [20] demonstrated that ZnO nanorods grown on bacterial cellulose exhibit strong antibacterial activity against *E. coli* and *S. aureus*, attributed to the uniform distribution of ZnO across the nanofiber surface. Similarly, in our previous work [59], chitin/ZnO nanohybrids showed high efficacy against both bacterial species, primarily due to the efficient stabilization of ZnO nanoparticles and the enhanced electrostatic interactions with bacterial cells. Across all systems, including the present CNF/ZnO nanohybrids, ROS generation and membrane disruption consistently appear as central antibacterial mechanisms, while Gram-positive *S. aureus* are found to be more susceptible than Gram-negative *E. coli*.

### 3.4. Evaluation of Antiviral Activity

The antiviral activity of c-CNF/ZnO and a-CNF/ZnO was evaluated against an enveloped bovine coronavirus (BCoV) and a non-enveloped feline calicivirus (FCV) under environmental conditions of 20 °C, with 24 h of contact time, and at various concentrations ranging between 10 and 500 µg/mL. The obtained results are summarized in Table 2. Prior to antiviral testing, both nanohybrids were examined for cytotoxicity against the MDBK and CRFK permissive cell lines. Neither nanohybrids exhibited detectable cytotoxicity above 0.5 log_10_ TCID_50_/mL, confirming suitable compatibility for downstream antiviral assays.

Both CNF/ZnO nanohybrids exhibited low antiviral activity (<1 log_10_ reduction) against both BCoV and FCV after 24 h of contact at 20 °C. However, at a concentration of 500 µg/mL, c-CNF/ZnO displayed stronger antiviral activity than a-CNF/ZnO against BCoV, achieving a 2.1 log_10_ reduction, corresponding to 99.21% antiviral efficacy. Against FCV, c-CNF/ZnO demonstrated enhanced potency at significantly lower concentrations: at 20 µg/mL, it outperformed the a-CNF/ZnO formulation tested at 500 µg/mL. From 100 µg/mL onward, c-CNF/ZnO achieved maximal antiviral activity, with a 4.9 log_10_ reduction (>99.99%), corresponding to a residual viral load below the detection limit.

These findings indicate that c-CNF/ZnO exhibits substantially higher antiviral activity than a-CNF/ZnO against both enveloped and non-enveloped viruses, though effective concentrations vary. Notably, the strongest antiviral effect was observed against FCV, a non-enveloped virus with a proteinaceous capsid. In contrast, only moderate viral reduction was achieved against BCoV, whose lipid-enriched envelope likely offers additional protection. Under identical experimental conditions, the antiviral activity of a-CNF/ZnO remained below 1 log_10_ reduction for both viral models.

Overall, these antiviral findings are consistent with mechanisms commonly associated with ZnO-based antiviral materials, including electrostatic disruption of viral surface structures, ROS-mediated damage to capsid proteins or lipid envelopes, and potential interference by released Zn^2+^ ions with viral integrity and infectivity [44,45]. The markedly higher activity of c-CNF/ZnO reflects the enhanced interactions afforded by its cationic surface, while the stronger effect against FCV suggests that non-enveloped viruses may be more vulnerable to oxidative and ionic disruption. Together, these mechanisms support the potential of c-CNF/ZnO hybrid as effective broad-spectrum antiviral agent.

### 3.5. In Vitro Evaluation of Cytotoxicity

**Figure 12:**
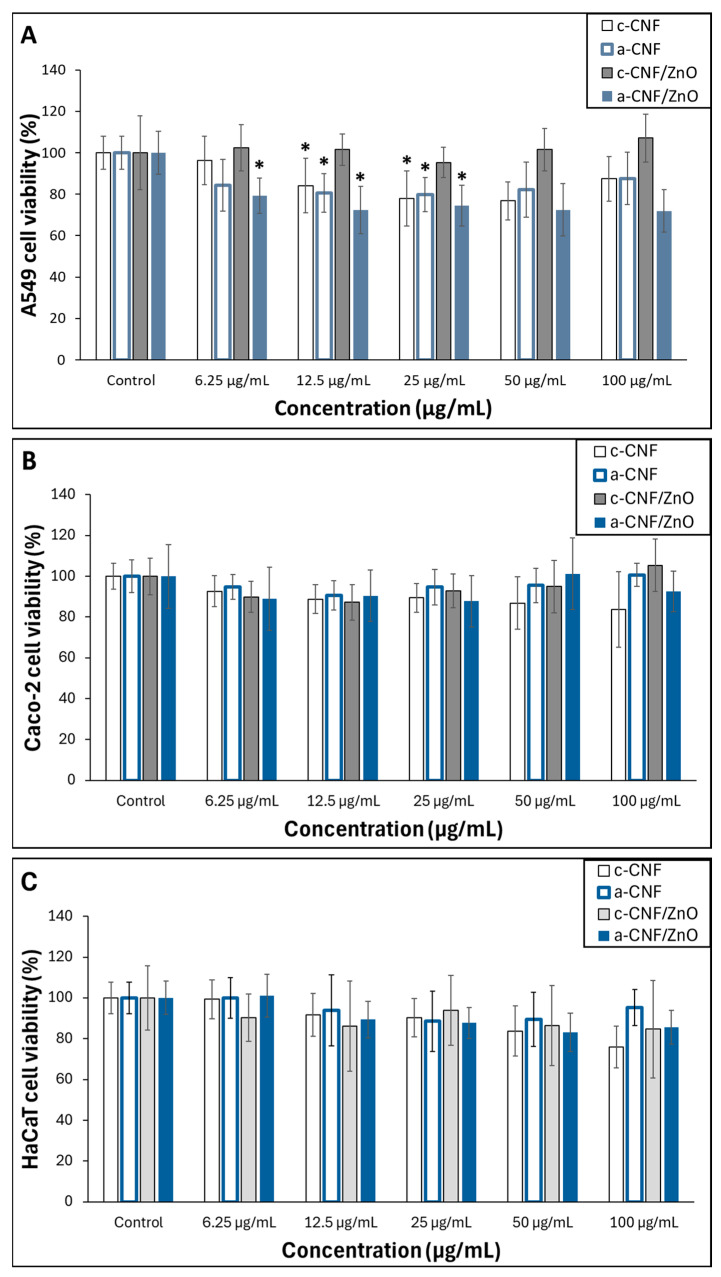
Cytotoxicity assessment of cellulose nanofiber-based formulations on human epithelial cell lines. Cell viability (%) of (**A**) A549, (**B**) Caco-2, and (**C**) HaCaT cells after 24 h exposure to different concentrations (6.25–100 µg/mL) of c-CNF, a-CNF, c-CNF/ZnO, and a-CNF/ZnO. Cells treated with PBS served as the negative control, and viability is expressed as a percentage relative to control (mean ± SD, *n* = 3). Statistical analysis was performed using one-way ANOVA followed by Tukey’s multiple comparison test, with differences considered significant at *p* ≤ 0.05, indicated as *; absence of annotation indicates no significance (*p* > 0.05).

The cytotoxicity of the parent cellulose nanofibers as well as of CNF/ZnO nanohybrids was evaluated against A549, CaCo-2, and HaCaT cells to assess their biocompatibility across lung, intestinal, and skin epithelial models. The cytotoxicity of the cellulose nanofiber-based formulations was evaluated in A549, Caco-2, and HaCaT epithelial cell lines following 24 h of exposure to concentrations ranging from 6.25 to 100 µg/mL (Figure 12).

Specifically, A549 cells after exposure to a-CNF and a-CNF/ZnO exhibited a moderate reduction in viability, particularly at higher concentrations (≥12.5 µg/mL, cell viability >70% and >80% for a-CNF/ZnO and a-CNF, respectively), indicating mild cytotoxicity. Interestingly, no cytotoxicity of c-CNF/ZnO was registered against A549 cells even at the highest tested concentration (100 μg/mL, cell viability ~100%). These data suggest that c- CNF/ZnO are biocompatible, even though it is known that A549 cells, being of pulmonary origin, are sensitive to nanoparticulate metal oxides, likely due to oxidative stress and mitochondrial disruption mechanisms.

Caco-2 and HaCaT cells showed high tolerance to a-CNF/ZnO and a-CNF treatments, with viability consistently above 90 and 80%, respectively, across all concentrations. As above, no statistically significant cytotoxicity was observed for c-CNF/ZnO and c-CNF. Overall, no clear concentration-dependent trend was observed, and the differences among formulations were minimal. Caco-2 cells are known for robust tight junctions and resistance to moderate oxidative stress, which may explain their relatively viability. The inherently robust barrier characteristics and oxidative stress resistance of Caco-2 cells likely contribute to this stable response. Thus, our results indicate excellent intestinal epithelial compatibility, even when ZnO is incorporated into the nanofibers and support the potential safe use of these nanofibers for oral or gastrointestinal-related applications. Regarding HaCaT skin epithelial cells, our results also indicate excellent dermal biocompatibility and suggest that cellulose-based nanofibers modified with ZnO are expected to be well-tolerated for skin applications.

Overall, across all three cell lines, c-CNF/ZnO demonstrates high biocompatibility, with no cytotoxic effects even at 100 µg/mL. The most sensitive model was A549 cells, possibly reflecting the higher susceptibility of pulmonary epithelia to a-CNF/ZnO, but no statistically significant toxicity was registered for c-CNF/ZnO. The lack of significant toxicity, especially of the c-CNF/ZnO hybrid, suggests that both the surface chemistry and charge coupled with in situ-formed ZnO NPs do not induce acute cytotoxicity under these experimental conditions. The results collectively support the potential safe biomedical or environmental application of these cellulose nanofiber/ZnO systems, although oxidative stress and long-term exposure studies would be valuable next steps.

### 3.6. LCA of Nanohybrids

#### 3.6.1. Life Cycle Assessment Results

**Figure 13:**
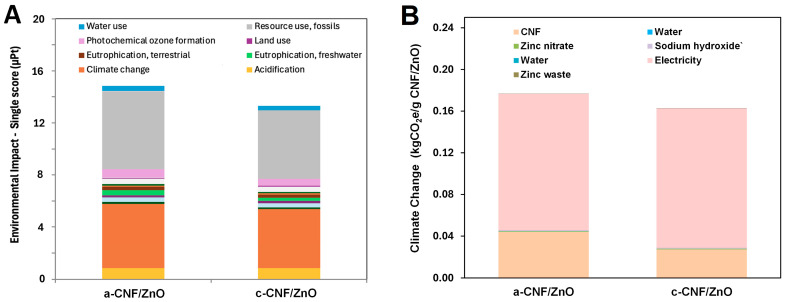
(**A**) Comparative environmental impact (single score). (**B**) Breakdown of climate change contributions of CNF/ZnO nanohybrids.

The environmental impact results for the CNF/ZnO nanohybrids are shown in Figure 13A,B). Figure 13A presents the total environmental impacts, expressed in micropoints. The results reveal that c-CNF/ZnO nanohybrid exhibit lower environmental burdens in the evaluated categories compared to their anionic counterparts (a-CNF/ZnO). The main indicators contributing to the total single score are fossil resource use, climate change, acidification, photochemical ozone formation, and water use, among others (Figure 13A).

The climate change assessment of the two nanohybrids indicates that electricity demand is the primary contributor to their environmental impact (Figure 13B), followed by emissions from upstream CNF production. These results highlight the importance of enhancing energy efficiency in pilot-scale operations, particularly in process stages requiring thermal input, such as heating to 70 °C for ZnO formation, and further optimizing both anionic and cationic functionalized CNF to mitigate critical hotspots during scale-up.

**Figure 14:**
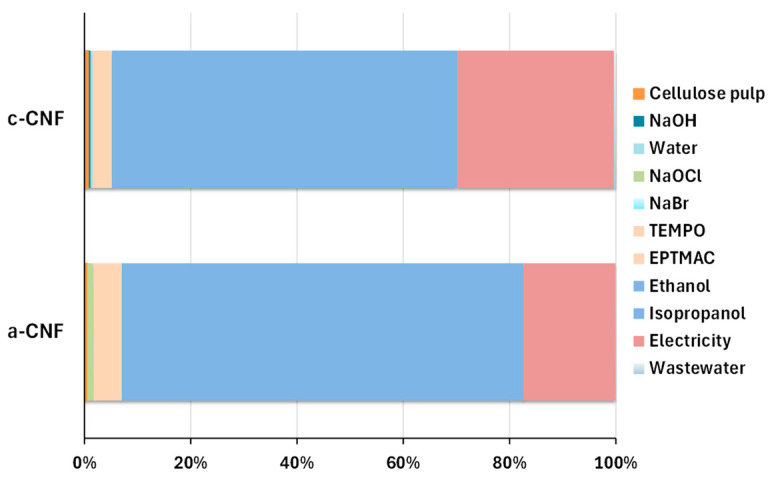
Comparative climate change impact distribution for CNF production.

As shown in Figure 14, the use of ethanol and isopropanol in CNF production contributes significantly to the overall impacts, but substantial reductions are expected at industrial scale, where high solvent recovery rates are typically achieved [93]. This industrial-scale optimization, combined with targeted energy efficiency measures or use of electricity from renewable sources, is anticipated to considerably lower the environmental footprint of nanohybrid production.

For the two evaluated nanohybrids in this work, the climate change values ranged from 167 to 178 kg CO_2_e/kg. For reference, the recent literature reports 800 kg CO_2_e/kg for TEMPO-oxidized CNF [94]. This result is even lower compared to high-value-added carbonaceous nanoparticles, such as reduced graphene oxide (produced via the Hummers method), which exhibit a climate change impact of 1060–2360 kg CO_2_e/kg [95]. It should be noted however that these values serve only as preliminary baseline references, as there is currently no available data in the literature for commercial bio-based nanohybrids or directly comparable materials with identical compositions as in this study. Therefore, this comparison should be considered indicative rather than definitive.

#### 3.6.2. Life Cycle Assessment Limitations

Bench-scale synthesis of nanofibers and then of nanohybrids exhibits limited throughput and operational inefficiencies, resulting in greater energy demand per functional unit, especially in energy-intensive impact categories such as climate change and depletion of fossil resources. Transitioning to pilot-scale or industrial manufacturing is anticipated to enhance process performance through improved heat and mass transfer, more consistent operational control, and lower standby energy consumption, ultimately yielding more representative life-cycle inventories.

LCA databases largely lack characterization factors for nanomaterial-specific emissions, and toxicological effects, which restricts the evaluation of potential hazards linked to nanoscale releases. In addition, no uncertainty assessment of the input parameters (e.g., employing Monte Carlo simulations) was performed; integrating these approaches in subsequent studies is suggested to ensure a more thorough evaluation.

One limitation of the nanocellulose LCA is that EPTMAC was represented using an Ecoinvent proxy, while TEMPO data were taken from life cycle inventory previously reported in the literature [96], which may introduce uncertainties in the LCA results.

## 4. Conclusions

In the present study, two sustainable cellulose/ZnO nanohybrids were successfully synthesized by employing bio-derived anionic and cationic cellulose nanofibers as templating matrices for the in situ hydrothermal growth of ZnO nanoparticles. Physicochemical characterization confirmed efficient cellulose functionalization and high-quality nanofibrillation, followed by environmentally sustainable synthesis of ZnO nanoparticles (≈10–20 nm) homogeneously dispersed within the nanofibers, in both hybrid systems. Both nanohybrids exhibited enhanced antibacterial activity against Gram (−) *E. coli* and Gram (+) *S. aureus* bacteria, with c-CNF/ZnO demonstrating superior efficacy, particularly against *S. aureus* (MIC/MBC = 50 μg/mL/100 μg/mL). ROS quantification assays and SEM imaging collectively demonstrated that bacterial inactivation proceeds through a multi-step mechanism involving electrostatic adhesion, ROS-induced oxidative stress, and membrane disruption. Antiviral assays further showed that c-CNF/ZnO achieved complete FCV inactivation at 100 µg/mL or above, and moderate activity against BCoV, whereas a-CNF/ZnO remained weakly active. These results highlight the decisive role of surface charge in nanomaterial–microbe interactions. Cytotoxicity studies in A549, Caco-2, and HaCaT cells confirmed high biocompatibility for both nanohybrids, with no significant loss of cell viability at the concentration range in which they exhibited antimicrobial activity (≤100 μg/mL). Additionally, Life Cycle Assessment indicated comparatively low environmental impact, with additional reductions expected upon solvent recovery and process optimization. Overall, this work demonstrates that cellulose–ZnO nanohybrids, and particularly the cationic one, offer a promising combination of sustainability, strong antimicrobial/antiviral efficacy, physicochemical stability, and cytocompatibility. Despite these promising results, several challenges remain, including the need to evaluate long-term stability, reusability, and antimicrobial durability under realistic application conditions, as well as to further investigate performance against a broader spectrum of pathogens. Future work will focus on optimizing ZnO loading and surface chemistry, scaling up the synthesis process, and validating performance in real-world environments, such as coatings, water treatment systems, packaging materials, and broader biomedical technologies. Overall, this work demonstrates that cellulose–ZnO nanohybrids, particularly the cationic system, offer a compelling combination of sustainability, strong antimicrobial and antiviral activity, physicochemical stability, and cytocompatibility, making them attractive candidates for next-generation antimicrobial materials.

## Figures and Tables

**Table 1:** MIC and MBC values of both cellulose nanofiber–ZnO nanohybrids as well as the parent anionic and cationic cellulose nanofibers against *E. coli* and *S. aureus* bacteria.

Samples	*E. coli*		*S. aureus*	
	MIC (μg/mL)	MBC (μg/mL)	MIC (μg/mL)	MBC (μg/mL)
a-CNF	>500	>500	>500	>500
c-CNF	>500	>500	>500	>500
a-CNF/ZnO	500	>500	100	300
c-CNF/ZnO	200	300	50	100

**Table 2:** Antiviral activity (R) and antiviral efficacy (%) of both CNF/ZnO nanohybrids against BCoV and FCV.

**c-CNF/ZnO**					
Bovine coronavirus			Feline Calicivirus		
µg/mL	R	%	µg/mL	R	%
500	2.1	99.21	500	4.8	99.99
200	0.8	84.15	200	4.9	>99.999
100	0.6	74.88	100	4.9	>99.999
20	0.7	80.05	20	2.5	99.68
10	0.5	68.38	10	0.9	87.41
**a-CNF/ZnO**					
Bovine coronavirus			Feline Calicivirus		
µg/mL	R	%	µg/mL	R	%
500	0.6	74.88	500	0.8	84.15
200	0.7	80.05	200	0.9	87.41
100	0.6	74.88	100	0.7	80.05
20	0.7	80.05	20	0.8	84.15
10	0.5	68.38	10	0.6	74.88

## Data Availability

The original contributions presented in this study are included in the article/Supplementary Material. Further inquiries can be directed to the corresponding author.

## Supplementary Materials

The following supporting information can be downloaded at: https://www.mdpi.com/article/10.3390/ma19020346/s1, Figure S1: Visible transmission spectra of a-CNF and c-CNF films in the wavelength range from 400 to 800 nm; Figure S2: Conductometric titration of 20 mL of c-CNF aqueous suspension (5 g/L) with 5 mM aqueous silver nitrate (AgNO_3_) solution; Table S1: Life Cyle Inventory, with data introduced in SimaPro software.

## Abbreviations

The following abbreviations are used in this manuscript:

a-CNF	Anionic cellulose nanofibers
AgNO_3_	Silver nitrate
ATR	Attenuated Total Reflection
BCoV	Bovine coronavirus
c-CNF	Cationic cellulose nanofibers
CFU	Colony Forming Units
CLSI	Clinical and Laboratory Standards Institute
CNF	Cellulose nanofibers
CO_2_e	Carbon Dioxide Equivalent
CRFK	Crandell-Rees Feline Kidney
DCF	2′,7′-Dichlorofluorescein
DCFH	2′,7′-Dichlorodihydrofluorescein
DMAO	7-Dimethylamino-4-methylcoumarin
DMEM	Dulbecco’s Modified Eagle Medium
*E. coli*	*Escherichia coli*
EDX	Energy-dispersive X-ray
EMEM	Eagle’s minimum essential medium
EPTMAC	(2,3-Epoxypropyl)trimethylammonium chloride
EthD-III	Ethidium Homodimer III
FBS	Fetal bovine serum
FCS	Fetal calf serum
FCV	Feline calicivirus
FTIR	Fourier Transform Infrared Spectroscopy
H_2_DCFDA	2′,7′-Dichlorodihydrofluorescein diacetate
HCl	Hydrochloride
HPH	High pressure homogenization
ICP-AES	Inductively Coupled Plasma–Atomic Emission Spectrometry
LB	Luria–Bertani medium
LCA	Life cycle analysis
LCI	Life Cycle Inventory
LCIA	Life cycle impact assessment
MBC	Minimum Bactericidal Concentration
MDBK	Bovine kidney epithelial
MIC	Minimum Inhibitory Concentration
MTT	Thiazolyl blue tetrazolium bromide
NaOH	Sodium hydroxide
NPs	Nanoparticles
PBS	Dulbecco’s phosphate-buffered saline
R	Reduction
ROS	Reactive oxygen species
*S. aureus*	*Staphylococcus aureus*
SEM	Scanning Electron Microscopy
STEM	Scanning Transmission Electron Microscopy
TCID_50_	50% Tissue culture infectious dose
TEM	Transmission Electron Microscopy
TEMPO	2,2,6,6-Tetramethylpiperidine-1-oxyl
TSB	Tryptic soy broth
UV–vis	Ultraviolet-visible spectroscopy
XRD	X-ray Diffraction
ZnO	Zinc oxide
