# Supplementary material for: Bio-Derived Cellulose Nanofibers for the Development Under Environmentally Assessed Conditions of Cellulose/ZnO Nanohybrids with Enhanced Biocompatibility and Antimicrobial Properties
Source: Materials (Basel). 2026 Jan 15;19(2):346. doi: 10.3390/ma19020346 (PMC12842912; doi:10.3390/ma19020346)
Supplement: Supplementary file 1 [file materials-19-00346-s001.zip › materials-4080161-supplementary.pdf]

Supporting information

Bio-derived Cellulose Nanofibers for the Development under Environmentally Assessed Conditions of Cellulose/ZnO Nanohybrids with Enhanced Biocompatibility and Antimicrobial Properties

Kyriaki Marina Lyra, Aggeliki Papavasiliou, Caroline Piffet, Lara Gumusboga, Jean-Michel Thomassin, Yana Marie, Alexandre Hoareau, Vincent Moulès, Javier Alcodori, Pau Camilleri Lledo, Albany Milena Lozano Násner, Jose Gallego, Elias Sakellis, Fotios K. Katsaros, Dimitris Tsiourvas and Zili Sideratou*

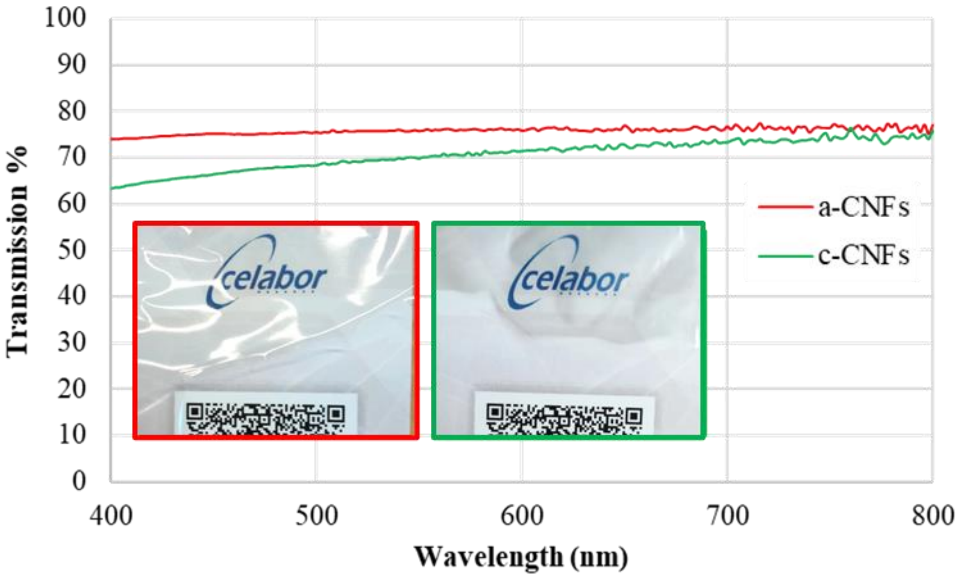

Figure S1. Visible transmission spectra of a-CNF and c-CNF films in the wavelength range from 400 to 800 nm.

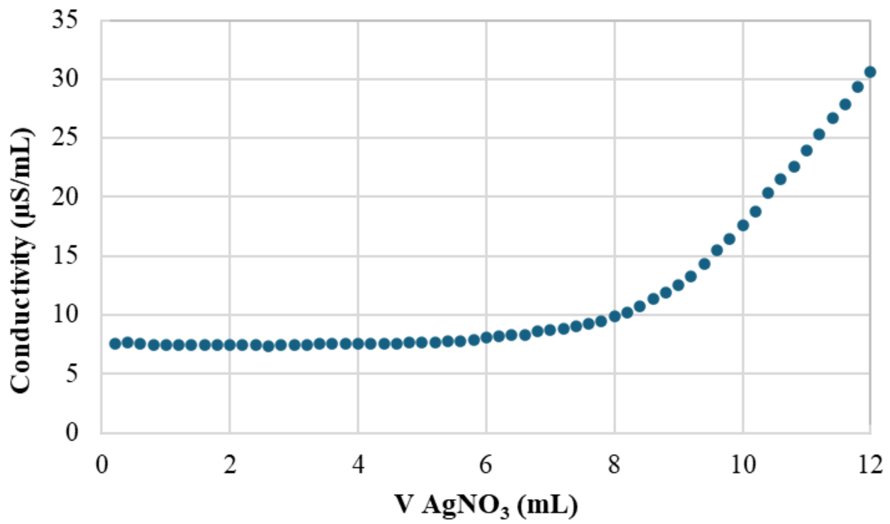

Figure S2. Conductometric titration of 20 mL of c-CNF aqueous suspension (5 g/L) with 5 mM aqueous silver nitrate ($AgNO_3$) solution.

Table S1. Life Cyle Inventory, with data introduced in SimaPro software

Materials	Units	a-CNF/ZnO	c-CNF/ZnO
Inputs			
a-CNF (1% aqueous dispersion)	g	340	0
c-CNF (1% aqueous dispersion)	g	0	300
Zn $(NO_3)_2 6H_2O$	g	3.8	3.6
NaOH (0.5N)	mL	2.5	2
Water (mL)	mL	380	380
Electricity (kWh)	kWh	1.86	1.85
Outputs			
Wastewater	mL	630	590
Waste Zn	g	0.53	0.45
